# New biological and immature morphological records of the masked chafer, *Cyclocephala paraguayensis*

**DOI:** 10.1093/jis/14.1.101

**Published:** 2014-08-01

**Authors:** Larissa Simões Corrêa de Albuquerque, Thamyrys Bezerra de Souza, Artur Campos Dália Maia, Luciana Iannuzzi

**Affiliations:** 1 Programa de Pós-graduação em Biologia Animal, Universidade Federal de Pernambuco, Av. Professor Moraes Rego s/n, Cidade Universitária, 50.670-901, Recife, Pernambuco, Brazil; 2 Departamento de Química Fundamental, Av. Jornalista Anibal Fernandes s/n, Cidade Universitária, 50.740-560, Recife, Pernambuco, Brazil; 3 Departamento de Zoologia, Universidade Federal de Pernambuco, Av. Professor Moraes Rego s/n, Cidade Universitária , 50.670-901, Recife, Pernambuco, Brazil

## Abstract

In order to obtain information on the biology of the masked chafer, *Cyclocephala paraguayensis* Arrow (Scarabaeidae: Dynastinae: Cyclocephalini), and its immature morphology, the beetle life cycle was studied under laboratory conditions. After field collection, adults were placed inside containers filled with soil obtained in the original capture to provide an oviposition site after mating ocurred. Eggs were collected daily and isolated for manipulation experiments and life cycle observations. Detailed information about the eggs, instars and life cycle duration, and morphological features of immature stages were noted and examined. Egg viability was higher in the “non-manipulated” batch. The complete ontogenic cycle of *C. paraguayensis* was 171 ± 11 days (n = 7). Despite the records of *Cyclocephala* being crop pests, reared larvae of *C. paraguayensis* thrived and developed into well-formed, fertile adults on an entirely saprophagous diet, indicating that they are not rhizophagous in the wild. The third instar can be distinguished from the other species mainly by the following unique characters: maximum width of the head capsule, distal antennal setae, and bifurcated setae on the raster.

## Introduction

The exceptionally diverse scarab beetles of the genus *Cyclocephala* Dejean, 1821 (Scarabaeidae: Dynastinae: Cyclocephalini) sum up to over 350 species ( Ratcliffe and Cave 2009 ), many of which are found in Brazil (Blackwelder 1944; Dechambre 1980 ; Endrödi 1985 ; Ratcliffe 1992 ; Andreazze 2001 ; Andreazze and Motta 2002 ; Ratcliffe 2008 ). Most adult *Cyclocephala* are phototactic and night-active ( Ratcliffe and Cave 2009 ). The latter trait can be associated to their role as pollinators of several species of night-blooming Neotropical angiosperms ( Gottsberger 1986 ; Bernhardt 2000 ). The larvae of *Cyclocephala* are edaph-ic and feed on dead plant matter and on the roots of grasses, sometimes leading to economically relevant crop damage ( Ritcher 1966 ). In some countries, like Colombia and the USA, some species are regarded as major agricultural pests ( Bran et al 2006 ). However, Deloya (1998) observed that larvae of *Cyclocephala lunulata* Burmeister fed on the leftovers of rice and sugarcane harvest in Mexico, strongly suggesting that they are saprophagous. In Brazil, the report of an exceptionally large infestation of white grubs from a unidentified species of *Cyclocephala* has not been linked to any sort of damage to the roots of wheat, barley, corn, or soybean (Gas sen 1989).

There is detailed information about the life cycles of only some species of *Cyclocephala.* These few studies are focused on species from temperate and subtropical and tropical regions. As such, in most tropical ecosystems, the unambiguous identification of larvae is hampered and pest management plans cannot be safely implemented ( Ramirez-Salinas et al. 2004 ). Exceptions include *C. lurida* Bland (previously referred to as *C immaculata* Olivier), *C. longula* (senior synonym of *C.abrupta), C. borealis,* and *C. pasadenae* ( Ritcher 1966 ); *C. signaticollis* Burmeister ( Morelli 1991 ; Mondino et al. 1997 ); *C. testacea* Burmeister ( Morelli and Auzugaray 1994 ); *C. fulgurata* Burmeister, *C lunulata* Burmeister, and *C. gregaria* Heney & Thaschenberg ( Bran et al. 2006 ); *C. forsteri* Endrödi ( Santos and Ávila 2007 ; Oliveira and Ávila 2011 ); *C comata* (Bates) ( García et al. 2009 ); *C verticalis* Burmeister ( Rodrigues et al. 2010 ; Coutinho et al. 2011 ); and *C. celata* ( Souza et al. 2013 ).

*Cyclocephala paraguayensis* Arrow exhibts a wide geographical range and is found from Central America all the way south to northern Argentina. In Brazil, it has been collected in all five macroregions (Endrodi 1985), being abundant in a faunistc survey (see Riehs 2006 ). Despite the lack of records proving this species is a pollinator, individuals were oberved visiting *Bauhinia curvula* Benth. (Leguminosae: Caesalpinoideae) in Brazilian cerrado ( Munin et al. 2008 ).

The purpose of this paper is to provide information on the biology of this species, notably on its life cycle and the morphology of immature stages.

## Materials and Methods

Adult *C paraguayensis* were captured in a 27 ha forest patch located on the grounds of Usi-na São José S/A sugarcane industry (USJ), municipality of Igarassu, Pernambuco State, in the northeastern coastal region of Brazil (7°49’16’’ to 7°50’54’’S; 34°49’26’’ to 35°00’35’’ O; altitude 113 m). On 4 March 2009, a light trap was installed to attract the beetles. From 18:00 to 21:00 hr, 250 W black-light and mercury vapour bulbs were deployed on opposite sides of a 2.5 x 2.0 m white sheet, stretched along the border of the forest fragment. The beetles were hand-picked as they landed upon the illuminated surface.

For both transportation and captivity rearing, adult *C. paraguayensis* individuals captured in the wild were placed in plastic containers with perforated lids (45 x 45 x 30 cm), filled to about %capacity with soil obtained *in situ.* Captivity maintenance followed protocols described for other species of dynastine scarab beetles ( McMonigle 2006 ; Lai and Hsin-Ping 2008 ). Three containers with 10-30 beetles each (1:1 male:female ratio) were kept under optimal ambient conditions of temperature and light. Soil moisture was controlled daily with the aid of a hand water sprayer. The beetles were fed apples and plantains, swapped every two days to avoid excessive fermentation and mold growth.

Oviposition was monitored daily. Recovered eggs were removed from the original containers and placed into new ones, also filled with soil obtained in the original capture site of the adults. They were split into two distinct experimental batches. In the first batch, the eggs were handled individually and weighed daily. These are hereafter referred to as “manipulated.” The eggs in the second batch were stored according to the date of oviposition and were not handled until hatching. These are referred to as “non-manipulated.” We have documented the following information for both batches during egg development: date and mode of oviposition, egg coloration, egg shape, date of larval eclosion, and egg viability, the latter following the method described by Rodrigues et al. (2010) .

After eclosion, the larvae were individually placed in plastic containers with perforated lids (30 x 30 x 30 cm). Each container was half-filled with feeding substrate for the larvae, which was kept optimally moist and periodically replenished. Composition of the substrate was adapted from formulas provided by McMonigle (2006) and Lai and Hsin-Ping (2008) and consisted of an even mix of finely pounded dead wood, ground leaf-litter, and commercially available humus (Gnúmus— Produção e Comércio de Húmus Ltda.). Larval development was monitored in five-day intervals, during which we recorded moltings, pupation, and date of emergence.

Four third-instar larvae and four pupae were dissected for anatomical observations. Prior to dissection, freshly sacrificed specimens were boiled in hot water for 3 minutes then fixed in Dietrich’s solution (600 mL of alcohol 96°, 300 mL of distilled water, 100 mL of 40% formaldehyde, and 20 mL of acetic acid) ( Marchiori et al. 2001 ). Afterwards, they were again boiled for 3 minutes in a 1:1 mixture of distilled water and Dietrich’s solution. The terminology used for anatomical descriptions of immature stages followed Böving (1936) and Ritcher (1966) . The images were taken through a digital camera attached to a Leica® M205 C stereomicroscope and processed using automontage software and Leica Application Suite (LAS) version 3.6.0 ( www.leica.com ). The images were edited with Adobe Illustrator CS5 and Adobe Photoshop CS3 ( www.adobe.com ).

## Results

### Biology

A total of 168 adult *Cyclocephala paraguayensis* were captured. Egg laying in captivity began after only three days of capture, when eggs were first observed and recovered from the substrate inside the rearing containers. Females oviposited at the bottom of the containers, and the eggs were laid individually. As has been described for several species of dynastine scarabs and other *Cyclocephala,* the females of *C. paraguayensis* protect their new eggs within a spherical clump of soil called the egg chamber (Rodrigues et al. 2010). Newly laid eggs have a thin membrane, and are consequently prone to dessication and abruption; the egg chamber provides extra protection ( Potter and Gordon 1984 ).

A total of 664 eggs were recovered from within the containers throughout the study, of which 245 were assigned to the “manipulated” batch and 419 to the “non-manipulated” batch. The complete ontogenic cycle of *C. paraguayensis* is 171 ± 11 days long (n = 7), similar to *C. celata* (≈164 days; Souza et al. 2013 ), but comparatively shorter than *C. verticalis* (≈228 days; Rodrigues et al. 2010 ) and *C. forsteri* (≈257 days; Coutinho et al. 2011 ).

Freshly oviposited *C. paraguayensis* eggs were oval-shaped and exhibited a milky white coloration, which gradually turned to brownish-yellow towards hatching. The egg mass at ovipostion was 1.0 ± 0.1 mg (n = 235), whereas prior to eclosion, the eggs were weighed at an average of 2.3 ± 0.6 mg (n = 67). Handling of the eggs seemed to have a negative effect over their development, leading to a considerable increase of the incubation time and decreased embryo viability. Egg development took an average of 18.7 ± 4.6 days (n = 16) within the batch of “manipulated” eggs, whereas within the “non-manipulated” batch, we observed an incubation time of 14.1 ± 1.5 days (n = 239). The viability of the eggs was recorded at 57% within the “non-manipulated” batch, against a much lower 6.5% within those in the “manipulated” batch.

During the ontogenic development of *C. paraguayensis* in captivity, we observed that the larval stage (notably, the second instar) exhibited the lowest mortality rate. Rodrigues et al. (2010) observed similar low mortality rates of second instars of *C. verticalis* reared in captivity. The highest mortality rate, 21.2%, was observed during the pupal stage. Overall, only 1% of *C. paraguayensis* eggs developed into new adults, a percentage significantly lower than that of *C. verticalis,* which was 9.1 % ( Rodrigues et al 2010 ). Souza et al. (2013) registered that 2% of *C. celata* eggs developed into adults.

The bodies of first instars of *C. paraguayensis* were translucent. At this stage, measurements of the maximum width of the head capsule averaged 1.12 ± 0.09 mm (n = 9). Newly hatched larvae took 42.2 ± 11.2 days (n = 112) before undergoing their first molting. The second instar was shorter in duration than the first instar (20 ± 6 days; n = 82). The average maximum width of the head capsule of second instars was 61.6% (1.81 ± 0.06 mm; n = 3), wider than that of first instars. The third instar was considerably longer than the previous one, lasting an average of 75.3 ± 22.8 days until prepupation (n = 33). Average greatest width of head capsule was measured at 2.87 ± 0.02 mm (n = 15), 58.6% wider than that of the previous instar. Comparatively, *C. celata* showed broader width of the head capsules and greater length between the immature stages ( Souza et al. in 2013 ).

**Figure 1.**
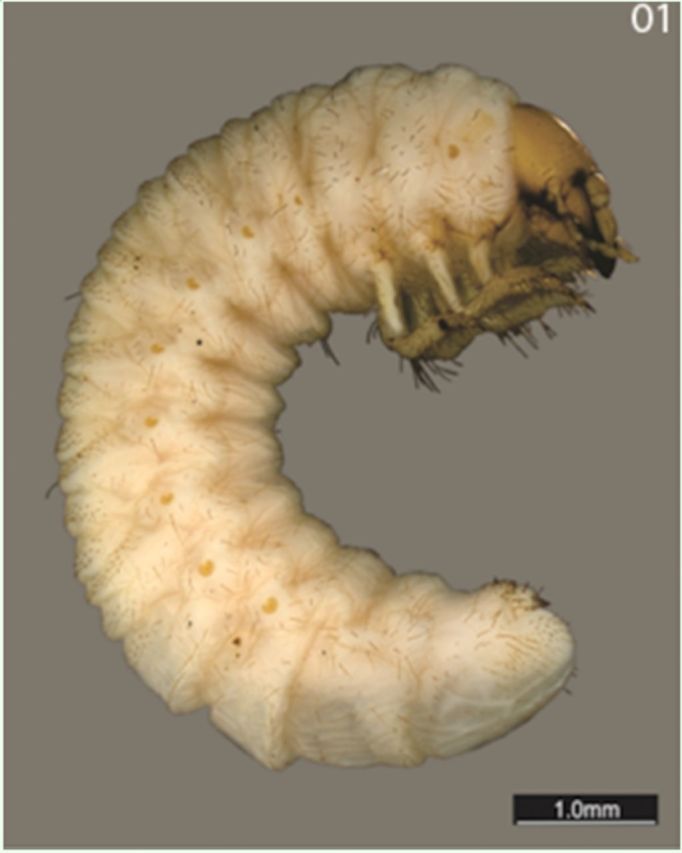
Prepupa of Cyclocephala paraguayensis. Habitus, lateral view. High quality figures are available online.

Towards prepupation, the integument of the third instars of *C. paraguayensis* turned from translucent to a milky-white coloration. Prepupae assumed a yellowish coloration, and the ventral surface of their last abdominal segment wrinkled ( Figure 1 ). The pupal stage took 11.4 ± 3.8 days (n = 7), shorter than *C. celata* (∽38 days, Souza et al. 2013 ). The average body length (from the head to the last abdominal segment) of the pupae was 12.11 ± 0.5 mm.

Despite numerous records of different species of *Cyclocephala* as crop pests ( Cherry 1985 ; Pardo-Locarno et al. 2005 ; Blanco and Hernández 2006 ; Santos and Ávila 2007 ; Villegas et al 2008 ; García et al. 2009 ), our observations suggest that such is not the case for *C. paraguayensis* . The fact that captive-reared larvae thrived and developed into well-formed, fertile adults on an entirely saprophagous diet might indicate that they are not rhizophagous in the wild. However, Neita-Moreno and Morón (2008) have reported that the larvae of *Ancognatha ustulata* Burmeister (Dynastinae: Cyclocephalini) may adopt an herbivorous diet when deprived of suitable dead plant matter. Further investigation about the feeding behavior of immature Cyclocephalini should yield more solid results.

### Third-instar larvae and pupae

**Body.** Cylindrical and c-shaped.

**Figures 2-9.**
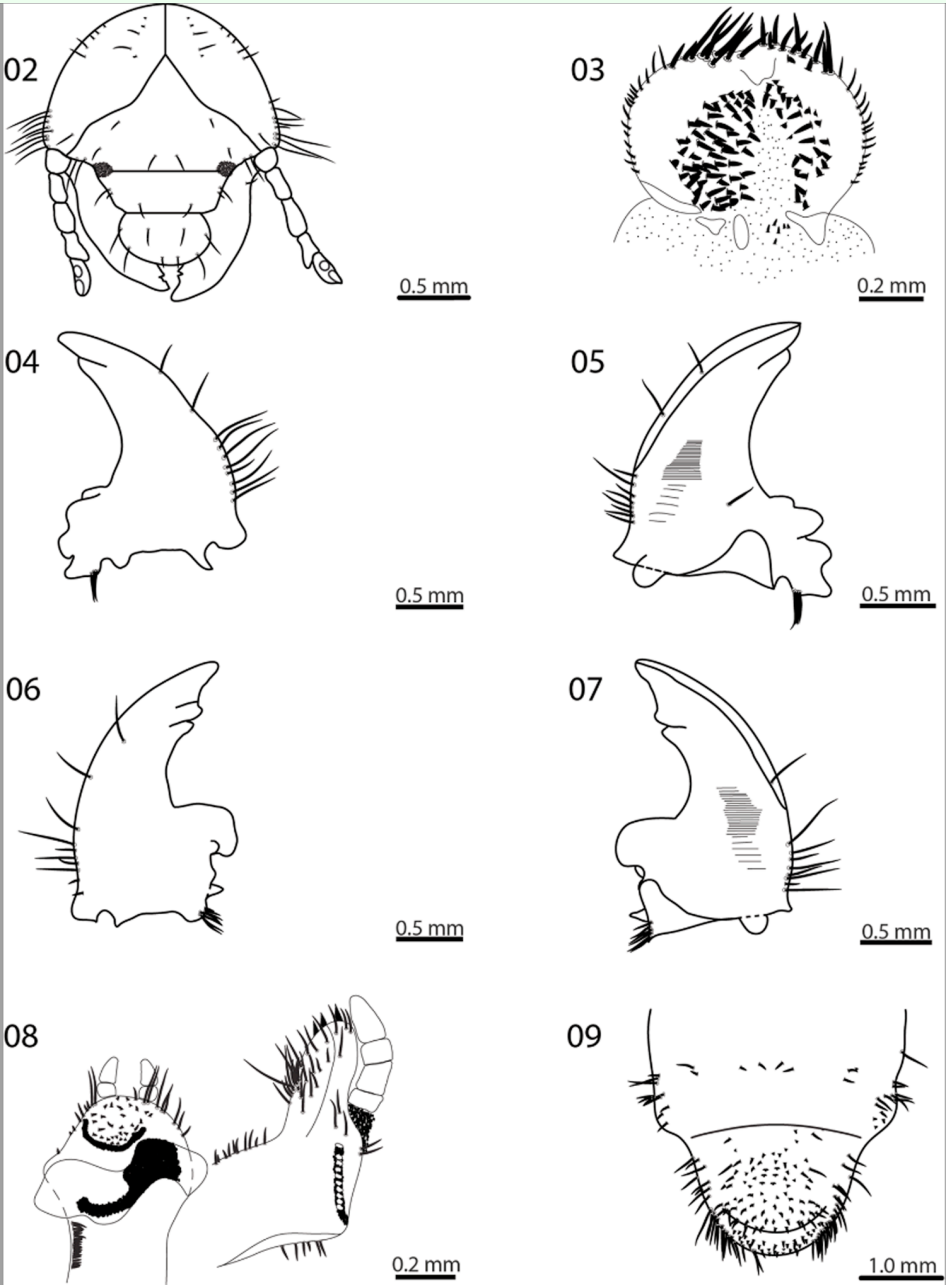
Morphology of the 3rd instar larva of Cyclocephala paraguayensis. 2 - Head, frontal view. 3- Epipharyx. 4-Right mandible, dorsal view. 5- Right mandible, ventral view. 6- Left mandible, dorsal view. 7- Left mandible, ventral view. 8-Maxila and hypopharyx. 9- Raster. High quality figures are available online.

**Head** ( Figure 2 ). Surface of head capsule yellowish, finely punctate. **Frons** . Each side with 2 posterior frontal setae, 2 anterior frontal setae, 3 anterior angle frontal setae, and 3 exterior frontal setae; cranial surface with 2 rows of dorsoepicranial setae with 5 setae aligned with the epicranial suture, 15 genal setae, and 3 paraocellar setae on each side. Clypeus trapezoidal with 2 exterior clypeal setae and 1 anterior clypeal seta on each side. Labrum cordi-form, slightly asymmetrical, and bilobed, with 1 anterior labral seta, 2 lateral setae, and 2 posterior setae on each side. Ocelli present, not pigmented. **Epipharynx** ( Figure 3 ). Haptomeral process notched, forming 2 teeth; right chaetoparia with 42 spine-like setae; left chaetoparia with 25 spine-like setae; acroparia each with 11-12 straight, long, thick setae; each acanthoparia with 13-15 short, curved, spine-like setae; pedium short, ovate. Dexiotorma narrow and elongate; laeotorma slightly shorter than dexiotorma and elongated toward apex; pternotorma rounded. **Mandibles** . Scissorial area and molar structures blackish. Ventral surface with elongate-oval stridulatory area with 6-7 thick ridges and about 30 slender ridges. Right mandible ( Figures 4 and 5 ): scissorial area with bladelike apical tooth (S1 + S2) and 1 rounded tooth (S3) after scissorial notch; caudolaterad of the scissorial notch is a single seta; scrobe with 1 slender, long seta; lateral face of mandible with 5-8 slender, long setae; calx large, basolateral setae absent; molar area with 3 wide, convex, ridged lobes (M1-3). Left mandible ( Figures 6 and 7 ): 3 incisive teeth (S1, S2 and S3); S1 and S2 separated by incisive notch; caudolaterad of the scissorial notch is a single seta; scrobe with 1 slender, long seta; lateral face of mandible with 8 slender, long setae; scissorial area with sinuate, bladelike apical tooth (S1+S2) and 1 rounded tooth (S3) after scissorial notch; scrobe with 1 slender, long seta; acia well-developed, sharp, without setae; basolateral setae absent; ventral process well-developed, rounded; brustia with 6-7 stout, long setae; molar area with 3 lobes, first molar lobe large. **Maxillae** ( Figure 8 ). Setose. Mala with visible suture. Galea with 1 falciform uncus. Lacinia with 3 falciform unci. Maxillary palpus 4-segmented, segments 1 and 3 subequal in length, segment 4 slightly longer than segment 2. Stridulatory area with 10 denticles. **Hypopharynx** ( Figure 8 ). Prominent scleroma, left side more developed. Right side with 22 short setae. More than 30 straight, slender setae caudolaterad of the bases of the 2-jointed labial palpi. The bare median portion of the glossa is surrounded by 30 stout setae. **Antennae** . 4-segmented. Distal portion of segment 3 prolonged, with sensory spot. Segment 4 piriform, with 4 sensory spots (2 dorsal, 2 ventral), bearing few setae distally.

**Thorax.** Prothoracic scleroma present at both sides. Legs well developed, setose. Prothoracic pair shorter than the mesothoracic, and the mesothoracic shorter than the metathoracic pair. Tarsal claw simple, brownish, with 1 basal seta and 1 prebasal seta.

**Raster** ( Figure 9 ). Teges with a broad patch of 32 setae surrounding two rows of 3–4 thick, bifurcated setae. Right barbula with 15 setae and left barbula with 12 setae. Anal slit transverse, curved, bearing 37 setae at the lower anal lip and numerous setae at the upper anal lip.

**Figures 10–11.**
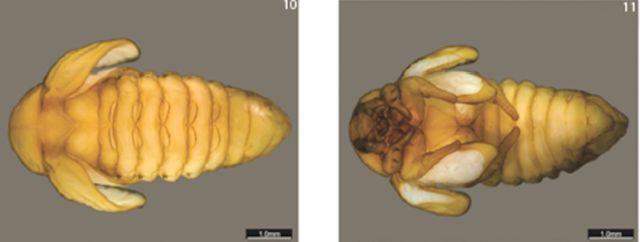
Pupae of Cyclocephala paraguayensis. 10- Habitus, dorsal view. 11- Habitus, ventral view. High quality figures are available online.

**Pupa** ( Figures 10-11 )

**Body.** Elongate, oval, exarate, and glabrous.

**Head.** Strongly deflected, mouthparts ventrally oriented; antennae, mandibles, palpi, and eye canthi recongnizable. Labrum and clypeus fused.

**Thorax.** Elytral tecae protect wing tecae, which are slightly bigger; wide subtriangular scutellum; evident ecdysial line, starting from prothorax all the way to the first abdominal segments.

**Abdomen.** Nine segments; segments I–V with pairs of dioneiform organs. Last tergites small, without urogomphus.

**Diagnosis.** Larvae are distinguished by the following unique characters: Frons: each side with 2 posterior frontal setae, 2 anterior frontal setae, 3 anterior angle frontal setae, and 3 exterior frontal setae. Epicranium: cranial surface with 3 rows of dorsoepicranial setae with 1, 2, and 5 setae aligned with the epicranial suture. Antennae: bearing few setae distally. Pupae: abdominal segments I-V with pairs of dioneiform organs.

### Key to larvae of the species *Cyclocephala* (modified of Ritcher 1966 and Souza et al. 2013 )

Antenna with 2 sensory spots on 4th antennomere .....................2 - Antenna with 4 sensory spots on 4th antennomere 5
(1). Cranial surface on each side with 7–10 dorsoepicranial setae .....................3 -Cranial surface on each side with 3–5 dorsoepicranial setae ...................... 4
(2). Maxilla with stridulatory area formed by combination of 1+13 teeth. Maximum width of head capsule 5.0 mm .………………… *C* . *testacea* Burmeister, 1847 - Maxilla with stridulatory area formed by combination of 1+10 teeth. Maximum width of head capsule < 5.0 mm *C. longula* LeConte, 1863
(2). Raster with 35 teges or more. Maximum width of head capsule 4.8 mm *C. comata* Bates, 1888 Raster with 25 teges or less. Maximum width of head capsule 3.9 mm *C. borealis* Arrow, 1911
(1). Frons on each side with 1 anterior frontal seta ......................6 - Frons on each side with 2 anterior frontal setae ..................... 7
(5). Frons on each side with 1 posterior frontal seta and cranial surface on each side with 5 dorsoepicranial setae ................. *C. signaticollis* Burmeister, 1847 -Frons on each side with 2 posterior frontal setae and cranial surface on each side with 8–10 dorsoepicranial setae 10
(5). Right mandible with 2 teeth in incisor area. Tarsal claws with 1 basal seta and 2 prebasal setae. Clypeus with 1 pair of setae in outer lateral margin on each side. Number of setae on right chaetoparia greater than on left chaetoparia ................. *C. celata*Dechambre, 1980 - Right mandible with 3 teeth in incisor area. Tarsal claws with 1 basal seta and 1 prebasal seta. Clypeus with 1 seta in outer lateral margin on each side. Number of setae on left chaetoparia greater than on right chaetoparia ..................................8
(7). Cranial surface on each side with 8–10 dorsoepicranial setae ..................................9 -Cranial surface on each side with 2 dorsoepicranial setae ................. *C. gregaria* Heyne & Taschenberg, 1907
(8) Maxilla with stridulatory area formed by combination of 1+9–10 teeth. Mandible with 21–28 dorsomolar setae. Raster with 25–30 teges ................. *C* . *fulgurata* Burmeister, 1847 -Maxilla with stridulatory area formed by combination of 1+7 teeth. Mandible with 9–11 dorsomolar setae. Raster with 20–25 teges ................. *C. lunulata* Burmeister, 1847
(7) Raster with 3 or 4 pairs of bifurcated setae ................. *C* . *paraguayensis* Arrow, 1913 -Raster without bifurcated setae *C* . *lurida* Bland, 1863
